# The matrix metalloproteinase inhibitor RS-130830 attenuates brain injury in experimental pneumococcal meningitis

**DOI:** 10.1186/s12974-015-0257-0

**Published:** 2015-03-04

**Authors:** Fabian D Liechti, Fabian Bächtold, Denis Grandgirard, David Leppert, Stephen L Leib

**Affiliations:** Neuroinfection Laboratory, Institute for Infectious Diseases, University of Bern, Friedbühlstr. 51, CH-3010 Bern, Switzerland; F. Hoffmann-La Roche Ltd., Pharmaceuticals Division, Grenzacherstrasse 183, CH-4070 Basel, Switzerland; Biology Division, Spiez Laboratory, Swiss Federal Office for Civil Protection, Austrasse, Spiez, CH-3700 Switzerland

**Keywords:** Matrix metalloproteinases, MMP inhibitor, Bacterial meningitis, *Streptococcus pneumoniae*, Neuroinfection

## Abstract

**Background:**

Pneumococcal meningitis (PM) is characterized by high mortality and morbidity including long-term neurofunctional deficits. Neuropathological correlates of these sequelae are apoptosis in the hippocampal dentate gyrus and necrosis in the cortex. Matrix metalloproteinases (MMPs) play a critical role in the pathophysiology of PM. RS-130830 (Ro-1130830, CTS-1027) is a potent partially selective inhibitor of MMPs of a second generation and has been evaluated in clinical trials as an anti-arthritis drug. It inhibits MMPs involved in acute inflammation but has low activity against MMP-1 (interstitial collagenase), MMP-7 (matrilysin) and tumour necrosis factor α converting enzyme (TACE).

**Methods:**

A well-established infant rat model of PM was used where live *Streptococcus pneumoniae* were injected intracisternally and antibiotic treatment with ceftriaxone was initiated 18 h post infection (hpi). Treatment with RS-130830 (75 mg/kg *bis in die* (bid) i.p., *n* = 40) was started at 3 hpi while control littermates received the vehicle (succinylated gelatine, *n* = 42).

**Results:**

Cortical necrosis was significantly attenuated in animals treated with RS-130830, while the extent of hippocampal apoptosis was not influenced. At 18 hpi, concentrations of interleukin (IL)-1β and IL-10 were significantly lower in the cerebrospinal fluid of treated animals compared to controls. RS-130830 significantly reduced weight loss and leukocyte counts in the cerebrospinal fluid of survivors of PM.

**Conclusion:**

This study identifies MMP inhibition, specifically with RS-130830, as an efficient strategy to attenuate disease severity and cortical brain injury in PM.

## Introduction

Pneumococcal meningitis (PM) is an acute disease characterized by high mortality and morbidity rates with persisting neurofunctional sequelae [1]. Neuropathological correlates of these long-term deficits include apoptosis in the hippocampus, a region critical for memory acquisition, and necrotic injury in the cortical areas, which are both observed in rodent models and human cases [2].

Matrix metalloproteinases (MMPs) have two distinct biological properties. As convertases, MMPs activate and release cytokines and their receptors while as proteinases they lyse extracellular matrix components. MMPs play therefore a critical role in different steps of the pathophysiology of PM, for example in inflammation, blood-brain barrier disruption and brain damage [3,4]. In the cerebrospinal fluid (CSF) of patients with bacterial meningitis, MMP-8 and -9 are upregulated and a high MMP-9 concentration is a risk factor to develop neurological sequelae [5]. Tumour necrosis factor (TNF) α, interleukin (IL)-1β and IL-6 are elevated during PM and part of a self-sustaining circle of inflammation, initiated by the invading pathogen [2,6,7].

In previous experimental studies of PM, several classes of MMP inhibitors successfully attenuated cortical injury [8]. RS-130830 (Ro-1130830; CTS-1027; hereafter named RS) is a potent hydroxamic acid MMP inhibitor of a second generation [9,10]. It was originally designed as a strong inhibitor of MMP-2, -3, -8, -9, -12, -13 and -14 (>1,000× more potent compared to MMP-1 and MMP-7), without inhibiting MMP-1 (interstitial collagenase), since inhibition of MMP-1 was believed to be associated with musculoskeletal side effects. This substrate profile differentiates RS from previously investigated broad-spectrum MMP inhibitors [9,11,12]. RS proved to be safe in clinical trials for osteoarthritis and to evaluate protective effects on liver fibrosis in hepatitis C virus patients [13-15]. The aim of the present study was to evaluate RS for its effect on inflammation and brain damage in an experimental model of PM.

## Findings

### Infant rat model of pneumococcal meningitis

A well-established infant rat model of PM was used as previously described [8,16]. All animal studies were approved by the Animal Care and Experimentation Committee of the Canton of Bern, Switzerland (license BE 100/11) and followed the Swiss national guidelines for the performance of animal experiments. A clinical isolate of *Streptococcus pneumoniae* (serotype 3) was prepared as described earlier [8]. Nursing Wistar rats (Charles River, Sulzfeld, Germany) weighing 25.6 ± 2.7 g were infected intracisternally on postnatal day 11 by injection of 10 μl saline containing log_10_ 5.6 ± 5.0 CFU/ml live *S. pneumoniae*. Intraperitoneal treatment with RS (75 mg/kg *bis in die* (bid), *n* = 40) or vehicle in control littermates (succinylated gelatine; *n* = 42) was initiated 3 h post infection (hpi) and repeated 15 min before injection of ceftriaxone at 18 and 27 hpi. PM was confirmed by quantitative analysis of bacterial titres in CSF at 18 hpi (RS: log_10_ 8.2 ± 8.1 CFU/ml, n = 37; control (Ctrl): log_10_ 8.4 ± 8.8 CFU/ml, *n* = 39; *P* = 0.52, Mann-Whitney test) [8]. Antibiotic therapy with ceftriaxone (Rocephine®, 2 × 100 mg/kg bid, intraperitoneally, Roche Pharma, Basel, Switzerland) was started at 18 hpi.

### Statistical analysis

Statistical analyses were performed by using GraphPad Prism software (Prism 6 for Windows, GraphPad Software Inc., San Diego, CA). If not stated otherwise, results are presented as mean values ± standard deviation. The D’Agostino and Pearson omnibus normality test was used to discriminate between parametric and non-parametric values. To compare data between two groups, an unpaired Student’s t-test was used for parametric data; otherwise, the Mann-Whitney test was applied. Mortality rates were calculated using log rank (Mantel-Cox) test for significance based on all infected animals and numbers of animals sacrificed due to ethical reasons (clinical score ≤2) or dying spontaneously. Fisher’s exact test was used to compare two outcomes (cortical injury and clinical scores). A two-tailed *P* value < 0.05 was considered statistically significant.

### Evaluation of clinical parameters in experimental PM

**Figure 1:**
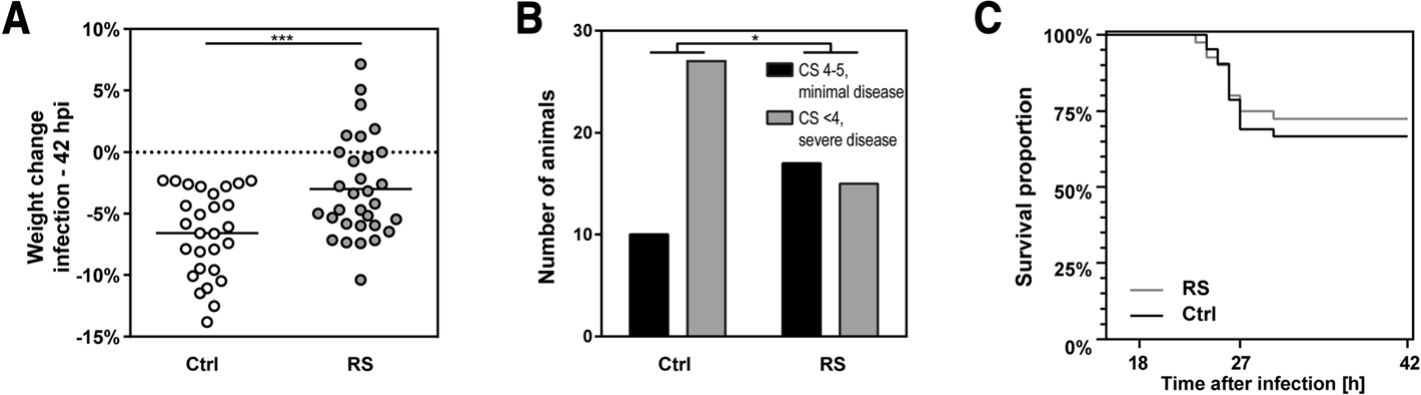
**Clinical parameters assessed during acute bacterial meningitis. (A)** Animals were weighed before intracisternal inoculation with pneumococci and before sacrificing at 42 hpi. Results are presented as percent difference from 0 to 42 hpi. Weight loss was attenuated in animals pre-treated with the MMP inhibitor (RS) compared to control animals (Ctrl). **(B)** At 27 hpi, a low-clinical score (CS < 4; severe disease) was observed in fewer (*P* < 0.05) animals treated with RS compared to littermates receiving vehicle. **(C)** Survival rates of animals treated with RS or vehicle were similar (hpi, hours post infection; CS, clinical score; *P < 0.05, Fisher’s exact test; ***P < 0.001, Student’s t-test).

At infection and at 18, 27 and 42 hpi, all animals were weighed and scored clinically by an investigator blinded to the experimental grouping (1 = coma; 2 = does not stand upright; 3 = stands upright within 30 s; 4 = minimal ambulatory activity, stands upright in less than 5 s; 5 = normal). Animals meeting ethical criteria for experimental abortion (clinical score ≤2) were euthanized with pentobarbital (Esconarkon, Streuli, Uznach, Switzerland, 150 mg/kg, intraperitoneally). During the acute phase of PM, that is between infection and 42 hpi, weight loss in animals treated with RS (−3.0 ± 4.1%, *n* = 31) was significantly attenuated compared to littermates (−6.6 ± 3.5%, *n* = 28; *P* < 0.001; Figure 1A). At the peak of disease (27 hpi), the mean clinical score was 3.7 (median 4.0, min. 2.0, max. 5.0, *n* = 32) in animals treated with RS and 3.4 in the control group (median 3.0, min. 2.0, max. 5.0, *n* = 37; *P* = 0.099, Mann-Whitney test). Only 15 of 32 animals (46.9%) in the RS group reached a clinical score lower than 4 compared to 27 of 37 animals (73.0%) in the control group (Fisher’s exact test, *P* = 0.047; Figure 1B). No differences between treatment groups were observed at 18 and 42 hpi. Survival rates were similar in animals treated with RS (29/40, 72.5%) and vehicle-treated littermates (28/42, 66.7%; log-rank test, *P* = 0.62; Figure 1C).

**Figure 2:**
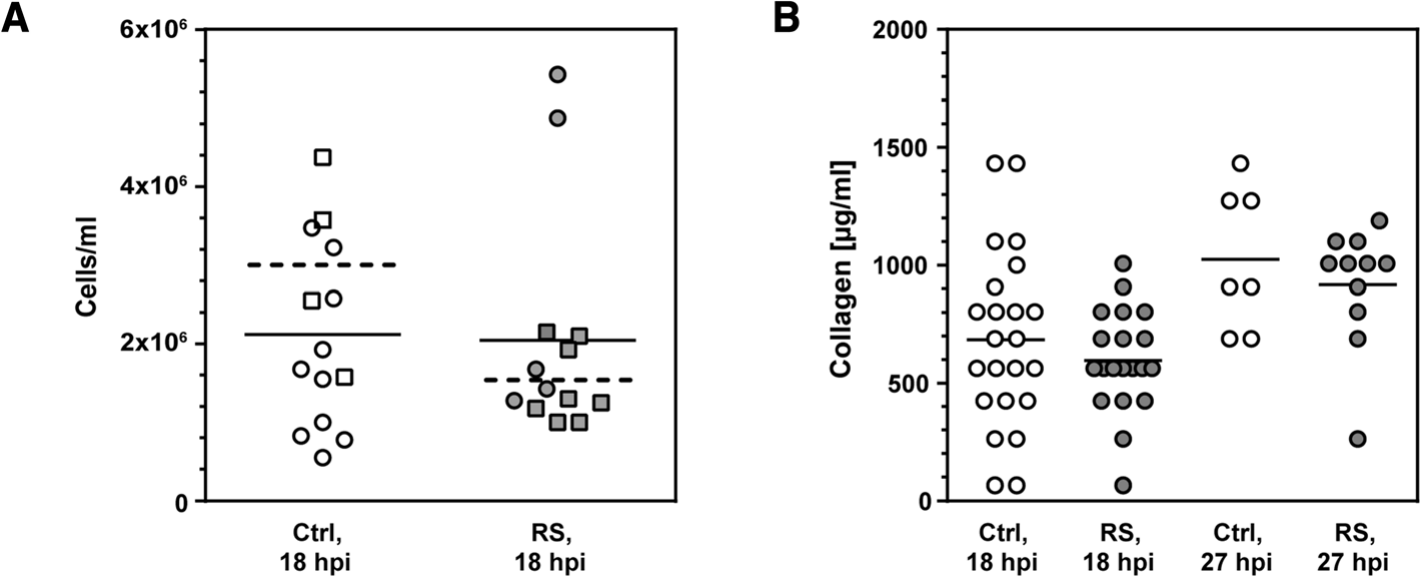
**Measurements in samples of cerebrospinal fluid. (A)** White blood cell count in cerebrospinal fluid samples obtained 18 h after infection (hours post infection (hpi)). No effect after pre-treatment with the MMP inhibitor (RS) was observed when compared to vehicle treated littermates (control (Ctrl)). Leukocyte counts were reduced after treatment with RS in the survivors of the disease (*P* < 0.05, Mann-Whitney test; squares). **(B)** Total collagen content was quantified at 18 and 27 hpi (optical density at 550 nm [OD_550_] was used as arbitrary unit). Concentrations were lower in animals treated with RS compared to control littermates, although this effect did not reach statistical significance (*P* = ns).

White blood cells (WBC) were counted in CSF at 18 hpi. Five microlitres of the non-centrifuged CSF was diluted 1:20 with 2% acetic acid. Cells were counted after 2 to 3 min in a Neubauer chamber. The difference between treatment groups did not reach statistical significance (RS: 2,044 ± 1,435 cells/μl, *n* = 13; vehicle: 2,118 ± 1,200 cells/μl, *n* = 14; *P* = 0.78; Figure 2A). However, in animals surviving until the endpoint at 42 hpi, leukocyte numbers at 18 hpi were significantly lower (*P* = 0.026) in RS-treated animals (1,488 ± 488 cells/μl; *n* = 8) compared to vehicle-treated animals (3,019 ± 1,218 cells/μl; *n* = 4).

### Inflammatory parameters and collagen content in the CSF

The concentration of selected cytokines involved in the pathophysiology of PM (TNF, IL-6, IL-1β, IL-10 and interferon γ [6]) was determined in the CSF using microsphere-based multiplex assays (Luminex Screening Assay, Rat Premixed Multi-Analyte Kit, R&D Systems, MN, USA). Five microlitres of CSF supernatant was diluted 1:10 with the provided assay buffer and measured as published previously [8]. An overall trend to decreased concentrations in the RS group compared to the control group was observed, reaching statistical significance for IL-1β and IL-10 at 18 hpi (Table 1). Peak concentrations for TNF, IL-6 and IL-1β were observed at 18 hpi, while IL-10 peaked at 27 hpi. At 42 hpi, except for IL-10, all cytokines were under or close to the detection limit provided by the manufacturer. Samples below detection were assigned the lowest detected values observed in the respective assay.

**Table 1:** **CSF cytokine concentrations in infected animals treated with RS or vehicle (Ctrl)**

	**Ctrl, 18 hpi (** ***n*** **= 11)**	**RS, 18 hpi (** ***n*** **= 10)**	***P*** **value** ^**b**^	**Ctrl, 27 hpi (** ***n*** **= 8)**	**RS, 27 hpi (** ***n*** **= 7)**	***P*** **value** ^**b**^
TNF [ng/ml]^a^	9.8 ± 4.0 (7.4, 5.3, 15.1)	8.9 ± 4.6 (8.0, 2.0, 17.1)	0.93	2.8 ± 2.3 (1.9, 0.9, 8.0)	1.8 ± 1.2 (1.1, 0.9, 4.3)	0.32
IL-6 [ng/ml]^a^	228 ± 67.4 (241, 127, 349)	173 ± 62.1 (166, 89.8, 299)	0.061	126 ± 96.0 (90.0, 27.1, 268)	120 ± 113 (60.5, 27.5, 317)	0.92
IL-1β [ng/ml]^a^	6.6 ± 2.7 (6.4, 2.2, 11.1)	3.6 ± 1.6 (3.8, 1.5, 6.3)	0.0079**	5.3 ± 3.8 (4.7, 0.6, 9.7)	3.7 ± 2.5 (3.1, 1.2, 8.0)	0.52
IL-10 [pg/ml]^a^	237 ± 118 (294, 30.0, 389)	70.2 ± 84.8 (30.0, 30.0, 243)	0.0051**	776 ± 806 (581, 30.0, 2,318)	500 ± 608 (30.0, 30.0, 1,297)	0.33
IFN-γ [ng/ml]^a^	7.0 ± 4.6 (8.0, 0.38, 13.3)	5.7 ± 5.4 (5.1, 0.38, 16.9)	0.48	8.9 ± 8.8 (7.3, 0.38, 20.0)	7.3 ± 7.0 (5.3, 0.38, 19.6)	0.86

^a^Values are mean ± standard deviation (median, minimum, maximum); ^b^Mann-Whitney U test. hpi, hours post infection; TNF, tumour necrosis factor α; IL, interleukin; IFN, interferon. **Statistically significant difference (*P* < 0.01).

Concentrations of collagen in CSF samples were assessed as an index of gelatinase/collagenase activity in the CNS as presented earlier [8]. A 10-μl CSF supernatant was diluted to a final volume of 100 μl (final concentration 6 M HCl) to hydrolyse the collagen to hydroxyproline at 95°C for 20 h. Of the centrifuged supernatant, 17.5 μl was diluted with 17.5 μl 4 M HCl and used in the Total Collagen Assay (QuickZyme Biosciences, Leiden, Netherlands). Absorption was measured at 550 nm. Collagen concentrations in CSF samples of RS-treated and non-treated animals were equal at 18 hpi (Ctrl: 684 ± 372 μg/ml, *n* = 23; RS: 596 ± 219 μg/ml, *n* = 20; *P* = 0.36; Figure 2B) and at 27 hpi (Ctrl: 1,024 ± 301 μg/ml, *n* = 7; RS: 916 ± 258 μg/ml, *n* = 11; *P* = 0.70), although a trend to lower CSF collagen concentrations in RS-treated animals was observed.

### Histomorphometrical analysis of brain damage

**Figure 3:**
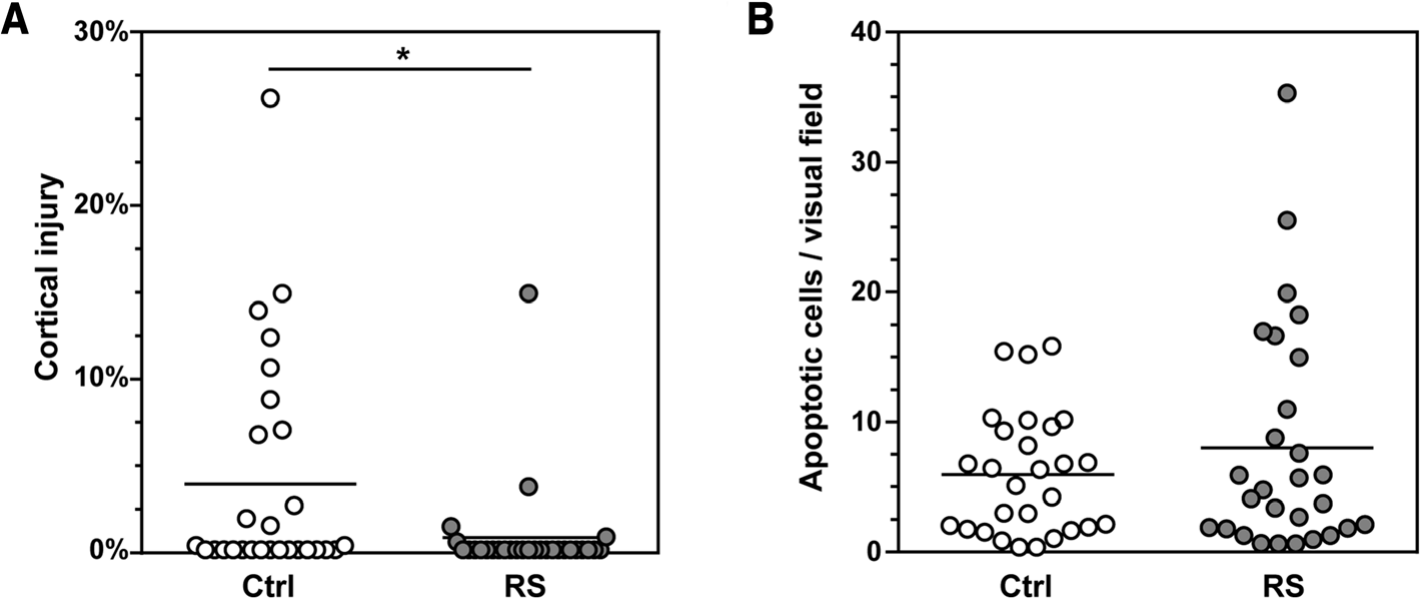
**Brain injury 42 h after infection. (A)** Statistically significant attenuation of cortical necrosis was seen in animals treated with the MMP inhibitor (RS) compared to littermates receiving the vehicle (control (Ctrl)), while no effect was observed on hippocampal apoptosis **(B)**. *P < 0.05 (Mann-Whitney test).

Coronal brain cryosections (45 μm-thick) of animals sacrificed at 42 hpi were stained with cresyl violet. Cortical damage was assessed systematically as previously published [8]. In controls, 11 of 28 animals (39.3%) showed substantial cortical injury (defined as ≥ 1% of the cortex) while this ratio was significantly reduced to 10.3% (3 of 29 animals) in littermates treated with RS (Fisher’s exact test, *P* = 0.015). In the control group, the mean necrotic area as percentage of the total cortical volume was 3.8% ± 6.5% (median 0.0%, min. 0.0%, max. 26.2%; *n* = 28) while this area was significantly reduced in the RS group to 0.7% ± 2.8% (median 0.0%, min. 0.0%, max. 14.8%, *n* = 29; *n* = 29; *P* = 0.012; Figure 3A). The number of WBC in CSF at 18 hpi correlated with the extent of cortical brain injury (*r* = 0.74, *P* = 0.0056, *n* = 12).

Apoptotic cells (histological features of condensed, fragmented dark nuclei; apoptotic bodies) were counted in the hippocampal dentate gyrus by a person blinded to the experimental grouping as published previously [8]. In animals receiving RS, 8.0 ± 8.8 apoptotic cells per visual field (c/f) were observed (*n* = 28) compared to 5.9 ± 4.7 c/f in control littermates (*n* = 28; *P* = 0.84; Figure 3B). In one animal of the RS group, apoptosis could not be quantified due to abundant pyknotic cells.

## Discussion

In the present study, RS attenuated cortical injury in infant rats with PM, which is in line with previous observations using broad-spectrum MMP inhibitors in PM [8,16-19]. RS combines high-oral availability and long half-time with an optimized substrate profile to target MMPs known to be upregulated in acute inflammation, while sparing MMP-1 and -7, and TNF converting enzyme (TACE), which sets it apart from other broad-spectrum MMP inhibitors and gives it a better clinical perspective [11,12,20]. These findings are limited in the present study to a pre-treatment regimen; however, an impact of the MMP inhibition, although probably inferior, could reasonably be expected in a more clinically relevant setting, that is starting RS application at 18 hpi. This would be in accordance with results of previous studies investigating other MMP inhibitors in the same experimental model [17].

Different from its beneficial effects on cortical injury, apoptosis in the hippocampal dentate gyrus was not influenced by RS. Metalloproteinase inhibitors targeting TACE have been shown in previous studies to attenuate this form of brain injury [8,17]. For RS, only a weak inhibition of TNF release has been reported with μM concentrations while the inhibiting concentrations (IC_50_) for most MMPs are × 1,000 lower (Roche, data on file, [9]). Therefore, metalloproteinase inhibitors with selective activity against TACE and MMPs upregulated in PM may be desirable.

MMP inhibitors prevent blood-brain barrier breakdown and reduce inflammatory parameters [21], witnessed in the present study by reduced CSF concentration of IL-1β and IL-10. We observed reduced leukocyte counts in CSF samples of surviving animals treated with RS. This finding may be related to attenuated cytokine release, that is IL-1β [6]. Attenuation of leukocyte recruitment prevents inflammatory processes, including release and activation of MMPs eventually leading to cortical injury [21,22]. This is the first observation showing a reduction of CSF WBC in animals treated with a MMP inhibitor [21], although the effect was exclusively detected in survivors and the number of samples available for this analysis was small due to the limited availability of CSF obtained by puncture in infant rats.

In the present study, hydroxyproline concentrations in the CSF were analysed as an index of CNS collagenase activity. Earlier studies also used gel zymography to determine CSF concentrations of MMP-9/-2; however, the interpretation of these results in the context of pharmacological MMP inhibition is difficult [8,23]. Collagen is also a substrate of MMP-1 [24], which may explain the weak effect of RS, which is inactive against MMP-1, on CSF collagen concentrations in the present study. The strong effect of RS on weight loss during acute PM, together with the mild effect on clinical score and survival, indicate relevant beneficial systemic effects of MMP inhibition in the present study. This proof-of-concept study in experimental PM demonstrates a beneficial effect of a partially selective second-generation MMP inhibitor that specifically targets MMPs upregulated in PM in attenuation of cortical brain injury.

## Abbreviations

bid: *bis in die*, twice daily
c/f: apoptotic cells per visual field
CSF: cerebrospinal fluid
Ctrl: controls (vehicle treated animals)
hpi: hours post infection
IL: interleukin
MMP: matrix metalloproteinases
PM: pneumococcal meningitis
RS: RS-130830
TACE: TNF converting enzyme
TNF: Tumour necrosis factor α
WBC: white blood cells
